# National Association of Dental Faculties

**Published:** 1899-08

**Authors:** 


### National Association of Dental Faculties.

This body held its annual session July 28th, and accomplished good work for our educational institutions. The influence of the meeting was effective for the general up-lifting of the profession. Some of the time, however, was devoted to the consideration of matters that were of but little interest to others than those immediately concerned. It seemed necessary that such subjects should have consideration. There were no papers read and but little consideration of subjects immediately pertaining to teaching, which we regard as unfortunate. Indeed this was one of the prime objects of the organization in the beginning, and it seems unfortunate that so much time must be devoted to matters of controversy and discipline. It is to be hoped for the next year that the faculties of our colleges will adhere so well to right doing that no person will have occasion to call in question their modes of procedure. "How good and how pleasant it is for brethren to dwell together in unity."

It is to be hoped that much time in the future will be devoted to the consideration of educational matters, modes of procedure, etc. It is desirable that there be several good papers, and that all will come well prepared for discussion upon the subjects that may be thus introduced.

That this body may grow and become stronger and stronger for good, and for the well-being of our educational institutions, is greatly to be desired.

